# Recurrent retroperitoneal sarcoma: beyond resectability toward a biology-based therapeutic framework

**DOI:** 10.3389/or.2026.1846525

**Published:** 2026-07-15

**Authors:** Dorian Yarih Garcia-Ortega, Montiel Jiménez Fuertes, Sergio Damian Quildrian, Nicolas A. Devaud, Javier Martin-Broto

**Affiliations:** 1 Surgical Oncology. Skin, Soft Tissue & Bone Tumors Department, National Cancer Institute, Mexico City, Mexico; 2 Unidad de Sarcomas, Servicio de Cirugía General y Digestiva, Hospital Universitario Fundación Jiménez Díaz, Madrid, Spain; 3 ⁠Sarcoma and Melanoma Unit, Soft Tissue Tumors Department, Angel H Roffo Institute of Oncology, University of Buenos Aires, Buenos Aires, Argentina; 4 Sarcoma and Melanoma Unit, General Surgery Department, Buenos Aires British Hospital, Buenos Aires, Argentina; 5 Cirugía Oncologica y HPB, Clínica Universidad de los Andes, Santiago, Chile; 6 Department of Medical Oncology, Fundacion Jimenez Diaz University Hospital, Madrid, Spain

**Keywords:** recurrence, retroperitoneal sarcoma, risk stratification, salvage surgery, therapeutic decision-making, tumor biology

## Abstract

Recurrent retroperitoneal sarcoma remains a significant therapeutic challenge. Recurrence does not always indicate treatment failure but reflects different tumor biologies with diverse implications for local and systemic management. This review proposes a biology-based framework for recurrent RPS that incorporates histology, disease-free interval, tumor growth kinetics, multifocality, resectability, functional reserve, and therapeutic proportionality. Histological subtype significantly influences recurrence patterns. Well-differentiated liposarcoma often follows a chronic local-regional course, where multiple surgeries can offer long-term control. In contrast, leiomyosarcoma more commonly shows early systemic spread, which diminishes the benefit of repeated local treatments. Disease-free interval and tumor growth rate are important dynamic indicators that improve prognosis and assist in timing treatment. Complete resection is essential for a meaningful survival benefit, but resectability alone does not justify surgery. Radiotherapy and systemic therapy serve complementary roles, mainly in local control, biological modulation, and palliation. Prognostic tools and nomograms aid in initial risk assessment, but are most valuable when combined with dynamic factors that reflect tumor behavior. Future advancements will rely on clinically relevant biomarkers, adaptive predictive models, personalized surveillance, recurrence-focused clinical trials, and shared decision-making based on meaningful patient benefits.

## Introduction

Retroperitoneal sarcoma (RPS) remains one of the most difficult diseases in surgical oncology because of its significant biological diversity, anatomical limitations that restrict wide margins, and a natural history characterized by locoregional recurrence. Even after curative-intent surgery at high-volume centers, recurrence occurs in 40%–60% of patients and is heavily influenced by histologic subtype, tumor grade, and the quality of the initial resection (1–3). Unlike most soft tissue sarcomas, RPS often requires long-term disease management rather than a single curative procedure (2,3).

Recurrence is not a uniform event but reflects tumor biology. Data from the Transatlantic/Collaborative RPS Working Group demonstrate that local recurrence is more common in liposarcoma, especially in well-differentiated and dedifferentiated subtypes, while leiomyosarcoma more frequently follows a metastatic pattern (1,4,5). These differences directly influence prognosis and the importance of locoregional versus systemic therapies.

With each recurrence, management becomes more complex, often requiring multivisceral or vascular resection and longer surgeries, which increase postoperative morbidity (6–10). Although salvage surgery can offer a survival benefit in selected patients, outcomes depend on disease-free intervals, multifocality, resectability, and tumor growth kinetics (6,7). Recurrence therefore shifts decision-making from solely aiming for anatomical eradication to assessing when intervention can meaningfully change the disease course.

Evidence remains limited and mainly comes from retrospective series, multicenter cohorts, and expert consensus, with little prospective data focusing on recurrent disease; landmark trials like STRASS addressed primary RPS (11,12). Meanwhile, management has shifted from binary treatment choices to personalized risk assessment using histology-specific nomograms, Sarculator, and dynamic variables such as growth rate (13–16). Centralization in specialized centers has also been consistently linked to improved survival, better local control, and lower perioperative morbidity (13,14).

Recurrence should therefore be understood as a stage of biologically persistent disease. In this review, we synthesize current data and expert opinion to propose a practical framework that integrates surgery, radiotherapy, and systemic therapy into an adaptive and personalized strategy (17–19). The key question is no longer only whether recurrent RPS can be resected but whether it should be resected.

### Definition and classification of recurrent disease in retroperitoneal sarcoma

Recurrent RPS should be viewed as a diverse clinical and biological spectrum rather than a single disease. Strictly, recurrence means the tumor reappears after a curative-intent resection with a confirmed disease-free period. This distinction separates true recurrence from the progression of remaining disease, which has important implications for prognosis and treatment (17,20,21). Recognizing this is crucial to avoid aggressive treatment in patients whose disease was never truly managed.

Anatomically, recurrence may occur as localized disease within the original retroperitoneal space, a pattern most commonly seen in well-differentiated and dedifferentiated liposarcoma, likely due to infiltrative growth and the challenge of achieving broad margins (1,4,5). It can also develop in a different retroperitoneal compartment without distant metastases, indicating more complex local spread (22). In contrast, metastatic recurrence—usually involving the lung or liver—is more typical of leiomyosarcoma and reflects hematogenous dissemination, which reduces the effectiveness of isolated local treatments (1,4,5). Peritoneal sarcomatosis is a distinct and particularly severe pattern, characterized by multiple peritoneal implants and limited opportunities for effective surgical intervention to alter the disease course (1,2,22).

Timing of recurrence also offers biological insights. Early recurrence, within 6 months of primary surgery, usually indicates occult residual disease or aggressive biology and is consistently linked to poor survival and limited benefit from salvage surgery (6,20,21). Recurrence at 6–12 months signifies an intermediate stage where surgery might still be justified, though less predictably. Late recurrence, beyond 12 months, more often reflects indolent biology and provides the most favorable scenario for repeat surgery aimed at extended control (6,20,21).

Multifocality, defined as multiple noncontiguous lesions within the retroperitoneum or abdominal cavity, can occur in all recurrence patterns. It is especially common in recurrent liposarcoma and is strongly linked to incomplete resection, repeated relapse, and poorer overall survival, making it one of the clearest clinical signs of aggressive biology and a practical limit to the curative potential of surgery (6,17,20,22).

The classification of recurrent RPS should go beyond mere description. Factors such as anatomic distribution, timing of recurrence (<6 months, 6–12 months, >12 months), multifocality, and association with prior surgery reflect the tumor’s biology. Their importance is not just in labeling the recurrence but in determining when intervention can change the disease’s course and when treatment should focus more on balance and symptom relief (6,17,20,21).

In clinical practice, the distinction between true local recurrence and progression of residual disease is best understood as a probabilistic classification rather than a fixed biological category. We propose the criteria summarized in Table 1 to improve reproducibility across centers and to reduce misclassification, including immortal time bias, in recurrence-focused studies. These pragmatic criteria may support multidisciplinary decision-making, outcomes reporting, and trial stratification. Very early recurrence should prompt concern for aggressive tumor biology or persistent disease; however, this interpretation must be applied cautiously in well-differentiated liposarcoma, where progression may remain indolent and radiographically subtle over time.

**TABLE 1:** Operational distinction between true recurrence and residual disease progression in recurrent RPS.

Criterion	True local recurrence	Progression of residual disease
Surgical status	Prior gross complete resection, usually R0/R1, with no known macroscopic residual disease	Prior R2 resection, debulking, aborted resection, or documented/suspected macroscopic residual disease
Early postoperative imaging	No measurable residual disease on baseline postoperative CT/MRI, preferably within 3–4 months	Persistent measurable or suspicious lesion on early postoperative CT/MRI.
Temporal pattern	New lesion after a documented disease-free interval, pragmatically >6 months	Lesion present from baseline imaging that enlarges or becomes more conspicuous, particularly within 3–6 months
Recommended reporting	Report as local recurrence and calculate disease-free interval from the date of complete gross resection	Report as progression of residual disease; do not assign a disease-free interval from surgery

### Tumor biology and histological-specific patterns of recurrence

Tumor biology is the primary factor influencing recurrence patterns in retroperitoneal sarcoma (RPS) and accounts for major differences among histological subtypes. Apart from tumor size or surgical technique, histology mainly determines whether recurrence is mostly local or systemic, thereby influencing the roles of surgery and systemic therapy (1,2,18,23).

Well-differentiated liposarcoma (WDLPS) is the clearest example of persistent locoregional disease. It carries a high risk of local recurrence, often exceeding 50%–60% with long-term follow-up, but a low rate of distant metastasis, usually below 10% (1,4,5). In this setting, recurrence does not necessarily indicate terminal disease, but rather a chronic relapsing course in which local control becomes the main challenge. For selected patients, repeated surgery may provide prolonged survival and durable disease control (1,5,18).

Dedifferentiated liposarcoma (DDLPS) holds an intermediate biological position. Although local recurrence is common, the risk of distant metastasis is notably higher, reaching 25%–30% in several series, and recurrences tend to happen earlier and behave more aggressively (1,4,5). This mixed pattern requires a combined approach: surgery remains crucial for locoregional control, but systemic surveillance and, in certain cases, medical therapy become more significant. In DDLPS, recurrence often indicates biological progression rather than just technical failure (1,2,18).

Leiomyosarcoma (LMS), by contrast, typically follows a mainly metastatic course. Local recurrence is less common, while early spread through the bloodstream, especially to the lungs and liver, characterizes its natural progression. In some studies, more than half of patients develop distant metastases within 10 years despite complete primary resection (1,4,5,24). As a result, salvage surgery has a more limited impact on overall survival, and systemic therapy becomes more important than in liposarcoma (1,2,24).

Less common subtypes, including solitary fibrous tumor, malignant peripheral nerve sheath tumor, and undifferentiated pleomorphic sarcoma, generally display more aggressive behavior and higher metastatic potential (2,23). In these tumors, local recurrence more often indicates advanced disease, and repeat surgery rarely provides the same prognostic value as in WDLPS. This highlights the importance of accurate histopathologic classification, ideally supported by expert review and molecular testing in specialized centers (17,23).

As summarized in Table 2, WDLPS functions as a chronic locoregional disease, DDLPS as a mixed locoregional-systemic condition, and LMS as a primarily metastatic disease. Recognizing these patterns offers a practical foundation for patient selection, surveillance, and treatment planning in recurrent RPS (1,2,17,23,24).

**TABLE 2:** Histology-specific patterns of recurrence and therapeutic implications in RPS.

Histology	Main recurrence pattern	Metastatic risk	Typical DFI	Role of salvage surgery	Role of systemic therapy
WDLPS	Locoregional	Very low (<10%)	Often long	Central; may allow repeated resections	Minimal role
DDLPS	Locoregional + distant	Intermediate (25%–30%)	Intermediate	Important if resectable	Selective
LMS	Distant metastasis	High (>50%)	Often shorter	Limited survival impact	Limited survival impact; selected cases only
SFT	Variable, often late	Variable	Long	Selective	Antiangiogenic therapy relevant
MPNST/UPS	Aggressive, systemic	High	Short	Rarely curative	Systemic priority

Recurrence should also be understood as a dynamic, evolutionary process. With each relapse, the tumor may be reshaped by clonal selection, prior surgery, radiation, systemic therapy, compartmental anatomy, and the local immune–stromal microenvironment. As a result, grade, dominant histologic pattern, cellularity, growth rate, multifocality, and recurrence interval may diverge from those observed at the primary operation. This spatiotemporal shift matters clinically: an indolent unifocal recurrence after a long interval and a rapidly expanding early multifocal recurrence are not simply different volumes of the same disease, but different biological states with different probabilities of complete resection, treatment response, and benefit from further local therapy (21,23).

### Natural history and prognosis of recurrence in retroperitoneal sarcoma

Several clinical variables help define prognosis in recurrent RPS, but their main value is biological rather than purely descriptive. To avoid overlap with operative decision-making, this section frames disease-free interval, multifocality, histology, and growth kinetics as markers of tumor behavior; the following section translates these same variables into surgical selection and treatment intent.

Disease-free interval is particularly informative. A long interval generally reflects a slower tumor tempo and is associated with better survival and a greater likelihood of durable benefit from salvage surgery. In contrast, early recurrence, especially within 6–12 months, often signals aggressive biology, occult residual disease, or rapid re-relapse, narrowing the window in which local treatment can meaningfully alter outcome (6,7,14,20,21).

Multifocality is another adverse biological marker. Multiple noncontiguous lesions are associated with lower rates of complete resection, earlier subsequent relapse, and poorer overall survival. Beyond its technical implications, multifocality suggests a more diffuse infiltrative process in which the capacity of surgery to change the natural course gradually diminishes (6,17,20,22).

Tumor growth rate adds a dynamic dimension to risk assessment. Unlike baseline clinicopathologic variables, growth kinetics observed on serial imaging reflect real-time disease behavior. Slow growth supports an indolent trajectory in which observation or delayed surgery may be reasonable, whereas rapid progression identifies patients in whom even technically complete resection is less likely to provide durable control (14,20).

Histology remains the framework within which these variables should be interpreted. Well-differentiated liposarcoma may recur repeatedly as a chronic locoregional disease, while leiomyosarcoma more often reflects systemic biology and dedifferentiated liposarcoma occupies an intermediate position, with both local and metastatic risks. The quality of initial surgery also shapes subsequent outcomes, as positive margins and incomplete resection are associated with earlier and more complex recurrence (1,4–8,17,18,23,24).

Taken together, these variables define two broad biological profiles. One is characterized by long disease-free interval, unifocal disease, slow growth, favorable histology, and realistic complete resection; in this setting, recurrence may remain amenable to repeated local control. The other is marked by early relapse, multifocality, rapid growth, aggressive histology, and limited resectability; here, recurrence is better understood as biologically dominant disease, and treatment should shift toward systemic therapy, palliation, surveillance, or symptom-directed care. Recurrence in RPS should therefore be viewed not only as treatment failure, but as the point at which tumor biology becomes clinically explicit (6,13,14,17,20).

### Evaluation of the patient with recurrent retroperitoneal sarcoma

Evaluation of RPS should be viewed as an integrated process that not only confirms relapse but also assesses whether active treatment can realistically alter the disease course. Imaging results, functional status, anticipated surgical complexity, and multidisciplinary input collectively define the biological context and determine the appropriateness of therapy.

Contrast-enhanced computed tomography (CT) of the chest, abdomen, and pelvis is the key method of assessment, identifying the extent of disease, multifocality, relationships to major organs and vessels, and the presence of distant metastases (2,19). In cases of recurrence, imaging offers both anatomical and biological insights: rapid progression, invasion of critical structures, or multiple implants strongly indicate aggressive disease and significantly lower the chance that surgery will offer meaningful benefit (2,17,19). Magnetic resonance imaging (MRI) is especially valuable for tumors involving the psoas, pelvis, spine, or complex muscular planes, and may help differentiate viable tumor from postoperative fibrosis in distorted surgical fields (2,23). FDG-PET can support selected cases but rarely alters management when macroscopic recurrence is already apparent (2,19).

Anatomical assessment should be complemented by evaluation of functional status. Salvage surgery for recurrent RPS often involves lengthy procedures and multivisceral resection, carrying a significant risk of major complications. Therefore, physiological reserve becomes an important prognostic factor (8–10). Conditions such as poor performance status, major cardiopulmonary comorbidities, renal dysfunction, or frailty significantly diminish the likelihood that aggressive surgery will provide proportional benefits (6,17).

Another important factor is anticipated surgical complexity. Recurrent disease develops in tissue altered by fibrosis, adhesions, and distorted anatomy, increasing the chances of vascular, bowel, and multivisceral resection (8–10,16). In published series, the oncologic value of surgery mainly depends on the likelihood of achieving a macroscopically complete resection. Incomplete surgery rarely improves survival and can expose patients to significant morbidity without a comparable benefit (7,25–27).

These considerations are most valuable when part of a specialized multidisciplinary evaluation. Data from TARPSWG and other observational studies consistently indicate that treatment at high-volume centers is linked to improved survival rates, reduced perioperative mortality, and better patient selection for salvage surgery (2,15–17). Multidisciplinary discussions should incorporate expert imaging review, specialized pathology, and coordinated input from surgical, medical, and radiation oncology to develop a cohesive treatment plan. Expert pathological review is particularly crucial because histologic reclassification can significantly change therapeutic priorities, especially in rare or aggressive subtypes (6,17,20).

In recurrent RPS, assessment defines the boundaries of meaningful intervention. Imaging, functional reserve, operative complexity, and multidisciplinary judgment must be integrated to determine whether treatment is likely to change the disease course or whether management should instead focus on proportionality and symptom control (2,6,17).

### Patient selection for salvage surgery in retroperitoneal sarcoma

In recurrent RPS, the central question is not whether surgery is technically feasible but whether it is oncologically appropriate. Patient selection should integrate tumor biology with the likelihood of complete macroscopic resection, expected morbidity, functional reserve, prior operative burden, nutritional status, and patient goals. Consistent with the TARPSWG framework, surgery should be considered only when complete macroscopic resection is realistically achievable and expected to provide meaningful oncologic benefit, either through survival gain or durable disease control (6,17,20). Thus, resectability is necessary but not sufficient; repeat surgery must also be biologically justified.

The clinical variables that define prognosis become most useful when they inform surgical judgment. A favorable candidate for salvage surgery is rarely defined by a single feature. More often, the decision rests on the convergence of a meaningful disease-free interval, unifocal or compartment-limited recurrence, slow growth kinetics, histology in which local control can plausibly influence outcome, realistic feasibility of complete macroscopic resection, preserved functional reserve, and patient goals aligned with the expected burden of treatment. In this sense, disease-free interval, multifocality, histology, and growth rate should not be treated simply as prognostic markers; they should be used to assess whether an operation is likely to achieve durable disease control rather than a short-lived anatomical clearance.

The threshold for surgery should rise as unfavorable features accumulate. Very early relapse, extensive multifocality, rapid growth, uncontrolled metastatic disease, diffuse peritoneal sarcomatosis, anticipated incomplete resection, frailty, poor performance status, or major comorbidities all reduce the likelihood that repeat surgery will provide proportional benefit. In these settings, the question is no longer whether a technically demanding resection can be attempted, but whether it can realistically alter the disease course in a way that justifies its biological and functional cost (6,17,20–22).

Histology modifies this threshold. In well-differentiated liposarcoma, repeated local treatment may remain appropriate when recurrence is localized, slow-growing, and completely resectable, given the disease’s often chronic locoregional course. In selected cases of dedifferentiated liposarcoma, salvage surgery may still be valuable when the aggressive component is anatomically limited and systemic risk appears controlled. By contrast, in leiomyosarcoma and other histologies with dominant metastatic potential, the expected survival benefit of salvage surgery is more limited, and surgery should be weighed carefully against systemic therapy, surveillance, or symptom-directed care (1,2,5,17,18,24).

Functional status remains the ultimate clinical criterion. Salvage procedures often require multivisceral resection, prolonged operative times, and are associated with high rates of major complications. Therefore, suitable candidates are typically limited to patients with preserved physiological reserve and adequate performance status (8–10,17). In frail patients or those with significant comorbidities, even technically complete resection may not confer proportional benefits.

Retrospective multicenter studies suggest that selected patients undergoing complete resection at first recurrence may achieve survival close to that of primary disease, although these findings are heavily influenced by selection bias, since surgery is usually reserved for patients with slow-growing, unifocal, and technically resectable disease (7,25–27). This warning should be clearly communicated during the multidisciplinary discussion: favorable postoperative results may reflect both the biology and physical condition of the selected patients and the therapeutic effect of the surgery (Table 3).

**TABLE 3:** Clinical factors supporting or discouraging salvage surgery in recurrent RPS.

Favorable factors	Unfavorable factors
DFI >12 months	DFI <6 months
Unifocal disease	Multifocal disease
Slow tumor growth rate	Rapid growth kinetics
Complete resection feasible	Incomplete resection anticipated
WDLPS/selected DDLPS	LMS with systemic disease
Good performance status	Frailty/major comorbidities
Managed at high-volume center	Non-specialized setting

Recognizing when not to operate is equally important; extensive multifocality, diffuse peritoneal sarcomatosis, a very short disease-free interval, uncontrolled metastatic disease, inability to achieve complete resection, and poor functional reserve are situations in which surgery rarely improves prognosis and may expose patients to substantial morbidity without a proportional oncologic benefit (6,17,20–22). In such scenarios, systemic therapy, palliative radiotherapy, active surveillance, or best supportive care should be presented as legitimate and ethically sound strategies, not merely fallback options after surgical exclusion.

**FIGURE 1:**
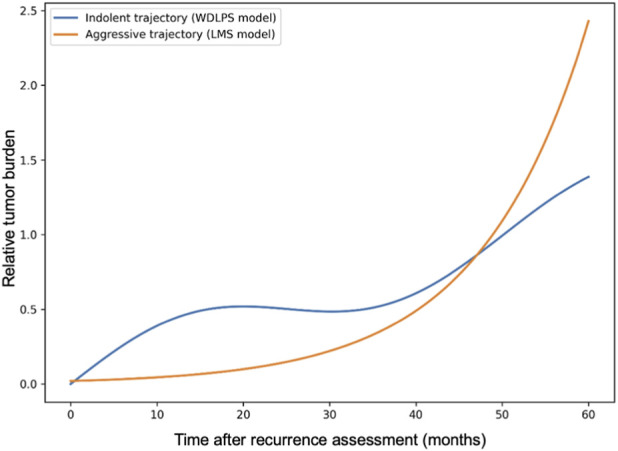
Conceptual Biological Trajectories in Recurrent Retroperitoneal Sarcoma. The figure contrasts an indolent, locally dominant pattern with a rapidly progressive, aggressive trajectory.

**FIGURE 2:**
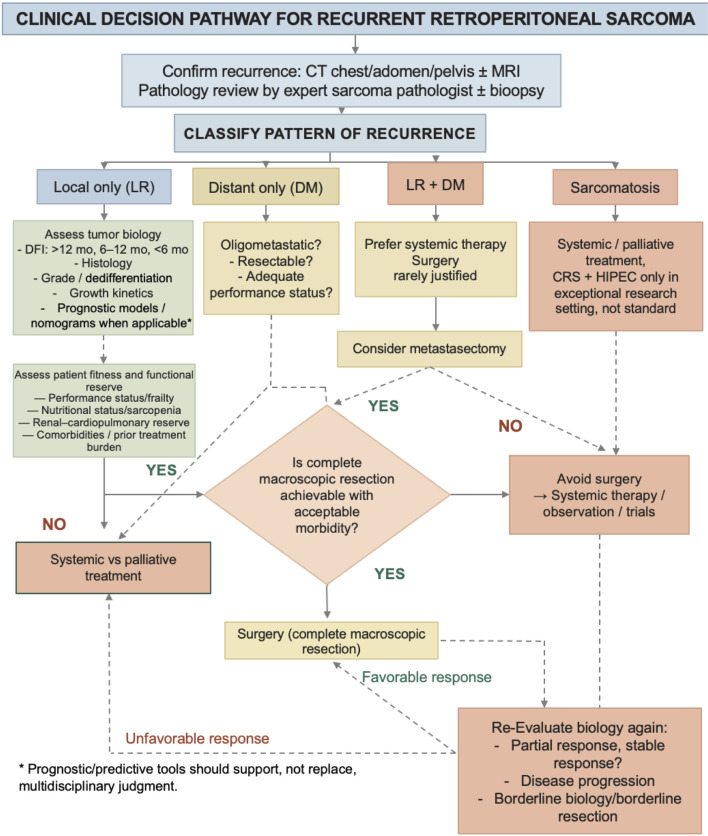
Proposed algorithm for the management of recurrent retroperitoneal sarcoma. LR, local recurrence; DM, distant metastasis; DFI, disease-free interval; PS, performance status; CRS, cytoreductive surgery; HIPEC, hyperthermic intraperitoneal chemotherapy.

Ultimately, the decision to proceed with salvage surgery for recurrent RPS should be based on favorable biology, realistic resectability, and sufficient functional reserve, rather than a strict algorithm (Figure 1). The main question is not whether the tumor can be removed, but whether surgery is likely to provide meaningful oncologic benefit. A structured, biology-driven decision pathway is outlined in Figure 2.

### Radiotherapy in recurrent retroperitoneal sarcoma

Radiotherapy in recurrent RPS plays a secondary role compared to surgery and should be viewed as a complementary, highly selective approach. Its effectiveness is limited by cumulative toxicity and the lack of solid prospective evidence. Unlike salvage surgery, which can improve survival in carefully chosen patients, radiotherapy for recurrence is mainly used to improve local control, alleviate symptoms, and sometimes enable complete resection later on (2,17,28).

Available evidence, mainly from retrospective institutional series, does not indicate a consistent overall survival benefit, although radiotherapy can offer meaningful locoregional control, especially in patients with unresectable disease or microscopic residual tumor after incomplete surgery (17,28). Its purpose is therefore not curative but strategic: to extend local control and maintain quality of life when surgery is not feasible or appropriate.

Modern techniques such as IMRT and VMAT have enhanced dose conformity and decreased exposure to nearby organs, including the bowel, kidneys, liver, and spinal cord, slightly broadening the use of radiotherapy in the retroperitoneum (11,17,28). Nevertheless, the therapeutic window stays narrow, and normal tissue tolerance remains a significant limitation, especially in previously irradiated patients.

Re-irradiation is especially controversial because many patients with recurrence have already undergone radiotherapy as part of their initial treatment. Additional doses of radiation significantly raise the risk of serious toxicity, including chronic enteritis, bowel obstruction, fistula, renal failure, and liver injury (2,17,28). Therefore, re-irradiation should not be standard practice but reserved for carefully selected cases in experienced centers with detailed dosimetric planning and a clearly defined clinical goal.

For isolated local recurrence after prior surgery, particularly in radiotherapy-naïve patients, conformal external-beam radiotherapy or intensity-modulated radiotherapy may be considered in specialized centers. However, reported local control beyond 12 months in 60%–70% of selected cases should be interpreted cautiously, as this evidence derives from retrospective, single-center experience rather than randomized data (17,28). Other focal approaches—including SABR/SBRT, percutaneous cryoablation, and radiofrequency or microwave ablation—may be reasonable in carefully selected patients with small, anatomically accessible, symptomatic, oligoprogressive, or surgically unsuitable lesions. Electroporation-based techniques remain investigational. Given the limited and heterogeneous evidence, often extrapolated from metastatic or non-retroperitoneal sarcoma cohorts, these modalities should be presented as MDT-selected adjuncts rather than substitutes for complete resection when surgery is feasible (29–31).

FDG-PET has been studied to differentiate fibrosis from active disease or to evaluate metabolic response, but its impact on management is usually limited, as it rarely alters treatment plans in cases of macroscopic recurrence (2,17,19). This underscores that radiotherapy decisions should rely on clinical and oncologic judgment rather than on isolated imaging findings.

A common mistake is to routinely recommend radiotherapy after incomplete salvage surgery to make up for less-than-ideal resection. Available data do not support this practice. Current consensus shows that radiotherapy is not a replacement for complete surgery and that using it without discrimination may lead to significant toxicity for patients without offering proportional oncologic benefits (17,28).

In recurrent RPS, radiotherapy should therefore not be applied reflexively, but only when aimed at a clearly defined and appropriate objective. Its purpose is specific: to enhance local control, support multimodal treatment in rare cases, or provide significant palliation when surgery is not suitable (2,17,28).

### Systemic treatment in recurrent retroperitoneal sarcoma

Systemic treatment in recurrent RPS should primarily be seen as a way to control the disease and provide palliation rather than a cure. Unlike salvage surgery, which may increase survival in carefully selected patients, systemic therapy usually does not alter the natural course of localized, potentially resectable recurrence. Its main purpose is in cases of metastatic, multifocal, unresectable, or rapidly progressing disease, where it can help alleviate symptoms and offer temporary tumor control (2,17,32).

Cytotoxic chemotherapy remains the mainstay of treatment for high-grade sarcomas. Doxorubicin, used alone or with ifosfamide, remains a standard first-line option for dedifferentiated liposarcoma, although response rates are modest and the survival benefit is limited (2,32,33). In cases of recurrence, its role is mainly palliative. Combination regimens may improve radiologic response but are associated with higher toxicity and do not consistently improve survival, underscoring the importance of careful patient selection (11,12,32). In certain patients, systemic therapy may also be considered when tumor shrinkage or stabilization could enable later surgery, especially if disease control persists beyond 6 months and the patient remains suitable for resection.

Although neoadjuvant “conversion” is often invoked in recurrent or advanced liposarcoma, its relevance in recurrent RPS remains limited by the lack of standardized benchmarks for conversion to resection and the intrinsically modest chemosensitivity of most liposarcoma subtypes. Across advanced liposarcoma, anthracycline-based therapy yields RECIST partial responses in only about 11%–12% of patients. Even in retroperitoneal DDLPS, where the dedifferentiated component may confer some treatment sensitivity, first-line chemotherapy yields partial responses in only a minority of cases, with most patients achieving stable disease or progressing. These data suggest that systemic therapy should not be viewed as a reliable route from unresectable recurrence to curative salvage surgery. Rather, its value lies in disease control, biological selection, and occasional cytoreduction in carefully selected DDLPS, with resectability ultimately determined by histology, growth kinetics, anatomical constraints, baseline operative feasibility, and MDT judgment (32,34,35).

Response to systemic therapy is highly dependent on histology. In well-differentiated liposarcoma, chemotherapy has minimal activity and is rarely justified (1,5,32). Dedifferentiated liposarcoma is somewhat more chemosensitive, but its benefit remains limited, usually restricting treatment to unresectable, metastatic, or rapidly progressive disease. In leiomyosarcoma, first-line treatment often includes doxorubicin plus trabectedin, while doxorubicin plus dacarbazine may be considered an alternative with less robust supporting evidence (36,37). Other regimens, including gemcitabine-docetaxel, gemcitabine-dacarbazine, and pazopanib, may offer meaningful control in later lines (2,32,33,37). Overall, these differences confirm that no single systemic strategy works for all recurrent RPS.

Targeted therapies have introduced a more biology-driven approach. In dedifferentiated liposarcoma, MDM2 and CDK4 amplification provide a rationale for subtype-specific inhibitors, which remain supported by limited clinical evidence but represent a logical option in a disease relatively resistant to conventional chemotherapy (18,38,39). In solitary fibrous tumor, antiangiogenic agents such as pazopanib, sunitinib, and sorafenib have shown notable activity and have largely replaced cytotoxic chemotherapy. These examples illustrate that the effectiveness of systemic treatment depends on close alignment with tumor biology.

Immune checkpoint blockade should be guided by histology rather than presented as a broadly applicable strategy for recurrent RPS. Although it is not a general standard of care, UPS has shown one of the most reproducible signals of activity among sarcoma subtypes: in SARC028, pembrolizumab produced responses in four of 10 patients with UPS, and Alliance A091401 suggested greater activity with nivolumab plus ipilimumab than with nivolumab alone, including responses in UPS. Neoadjuvant data also point to marked histology-specific immune sensitivity, with substantially higher median pathologic hyalinization in UPS than in retroperitoneal DDLPS. Thus, for retroperitoneal UPS or highly inflamed sarcoma phenotypes, checkpoint blockade is best discussed as a trial-preferred or biomarker-informed option, rather than dismissed as uniformly ineffective (40–42).

In selected cases, systemic therapy may also serve as a biological selection tool. In locally advanced or marginally resectable recurrence, disease stabilization or partial response may help identify tumors with less aggressive kinetics and clarify whether later surgery remains appropriate. However, this approach remains investigational, supported by limited evidence and significant selection bias, and should be confined to specialized centers. Its primary goals are disease stabilization, symptom relief, and preservation of quality of life, since complete responses are rare and durable stabilization is clinically meaningful. Importantly, systemic therapy should not be viewed as a substitute for complete surgery or as a way to compensate for incomplete resection, as current evidence does not support either assumption (2,17,20).

Overall, systemic therapy in recurrent RPS should be used selectively as a tool for biological control rather than a cure. Its value depends on careful integration of histology, recurrence pattern, tumor kinetics, and patient fitness within a specialized multidisciplinary framework.

### Prognostic models and predictive tools in retroperitoneal sarcoma recurrence

Prognostic models are increasingly relevant in recurrent RPS because they translate clinical judgment into structured, reproducible risk estimates. Their role should be clearly distinguished from narrative prognostic assessment. Rather than redescribing disease-free interval, multifocality, histology, or growth kinetics, predictive tools should be used to synthesize these variables into estimates that support MDT discussion, patient counseling, and therapeutic proportionality. In this setting, the value of a model is not only statistical; it lies in making complex clinical reasoning more transparent and less reliant on individual interpretation.

Histology-specific nomograms, particularly those developed from TARPSWG multicenter cohorts, remain the primary reference standard for RPS. They formalize a central principle of recurrent disease: prognosis is shaped by tumor biology and histology, not by anatomy or resectability alone. These tools can estimate the probability of survival, recurrence, and disease progression under defined clinical conditions and may be especially useful when the MDT must decide whether technically feasible surgery is also oncologically justified (6,13,17). When predicted outcomes remain poor despite apparent complete resectability, the threshold for repeat surgery should be raised. In that context, the model does not deny surgery but forces the team to define more explicitly what benefit is realistically expected.

Sarculator pragmatically extends this approach by generating individualized risk estimates from clinicopathologic variables and improving consistency in risk communication (13). However, its use in recurrent RPS should be approached cautiously. Most available tools were developed primarily for primary or mixed sarcoma populations and do not fully capture recurrence-specific features, including evolving tumor burden, multifocality, prior treatment exposure, disease-free interval, and changes in growth behavior over time (6,13,14,20). Sarculator may therefore provide a useful baseline estimate, but it should not be used as a stand-alone decision system for recurrent disease.

The main limitation of current prognostic tools is that they are largely static, whereas recurrent RPS is dynamic. A model calculated at a single time point may underestimate or overestimate risk if the disease subsequently changes in distribution, growth rate, treatment response, or histologic behavior. For this reason, prognostic assessment in recurrent RPS should be iterative. Baseline estimates should be revisited after interval imaging, systemic therapy, symptom evolution, or any major change in performance status. This is particularly important for patients being observed before surgery or treated with systemic therapy as a biological selection strategy.

A practical framework would use prognostic tools in three steps. First, TARPSWG nomograms or Sarculator can establish baseline risk. Second, recurrence-specific features such as anatomical pattern, resectability, disease-free interval, and multifocality can refine that estimate. Third, dynamic information from serial imaging, treatment response, and functional trajectory should recalibrate the decision before committing to surgery, radiotherapy, systemic therapy, surveillance, or palliation. Used this way, models do not replace clinical judgment; they discipline it.

For research, these tools also offer a means to improve patient stratification in a disease where prospective trials are difficult. Future recurrence-focused studies should incorporate histology-specific risk models, standardized definitions of recurrence versus residual disease progression, growth kinetics, treatment response, and patient-centered endpoints. External validation, calibration, and decision-curve analysis will be essential before any model is considered actionable in routine practice. Until then, prognostic models should be viewed as decision-support tools, not as treatment algorithms.

In recurrent RPS, the best use of predictive tools is therefore integrative rather than deterministic. They should help clinicians ask sharper questions: Is the patient’s risk profile compatible with meaningful benefit? Does the observed biology support intervention now, later, or not at all? Is the expected benefit proportional to the functional and biological cost of treatment? When applied in this way, prognostic models make decision-making more explicit, reproducible, and ethically defensible in a setting where both overtreatment and undertreatment carry substantial consequences (6,13,14,20).

### Quality of life, shared decision making, and ethical proportionality

Repeat surgery for recurrent RPS highlights the balance between oncologic benefit and cumulative biological costs. Unlike primary surgery, which is usually performed with curative intent, salvage surgery is performed in the context of ongoing disease, where each intervention adds to the burden of prior operations, multivisceral resections, and locoregional or systemic treatments (10,17,20). In this setting, quality of life is not secondary but central to assessing whether surgery is justified.

The surgical burden accumulates over time. Each reoperation increases technical difficulty, extends operative time, and raises the risk of vascular or bowel resection and major complications (8–10). Although some patients may have longer survival after multiple complete resections, this benefit must be weighed against the morbidity that impacts independence, nutrition, physical function, and daily life. Survival should therefore be evaluated alongside the conditions under which it is achieved.

This supports integrating patient-reported outcomes, including the EORTC QLQ-C30 and sarcoma-specific modules, to more objectively evaluate the value of salvage surgery. Recurrence-focused studies should also include endpoints such as time spent outside the hospital, time free from major complications, and preservation of independence, since meaningful benefits depend not only on survival duration but also on its quality.

Even after primary surgery, quality of life often declines and may only improve partially; following salvage surgery, recovery tends to be slower and less complete (10,43). Assuming normalization after multiple procedures is therefore not evidence-based and could lead to unrealistic expectations.

Success in recurrent RPS must therefore be redefined. It cannot be measured solely by complete resection or prolonged survival, but by the balance between disease control, functional preservation, and treatment burden (3,17,20). A technically successful operation resulting in severe dependence, recurrent hospitalization, or irreversible functional decline may offer limited oncologic value, even if temporary disease control is achieved.

Clear communication is essential. Patients should understand that salvage surgery is rarely curative and that disease control is often temporary (17,20). Potential long-term effects—including ostomy, nutritional issues, chronic pain, or ongoing functional limitations—should be discussed openly. Without this, consent may become procedural rather than genuinely informed (17,44).

The ethical principle guiding these decisions is therapeutic proportionality. Surgery is justified only when the expected oncologic benefit reasonably outweighs biological, functional, and emotional costs (3,17,20). When the benefit is uncertain and the burden is high, choosing not to operate is not abandonment but an active and ethically sound decision. Shared decision-making is therefore essential, and options such as surveillance, systemic therapy, palliative radiotherapy, or best supportive care should be presented as valid choices (17,44).

Repeat surgery is justified only when there is a realistic chance of oncologic benefit, an acceptable expectation of functional preservation, and clear patient understanding of treatment implications (3,17,20). Otherwise, surgery risks becoming disproportionately aggressive care.

### Current controversies in recurrent retroperitoneal sarcoma disease

Controversies in recurrent RPS stem more from the gap between technical feasibility and meaningful oncologic benefit than from a lack of treatment options. The main question is not what can be done, but what should be done when the expected benefit is uncertain and biological costs may be high (6,17,20).

One major controversy involves surgery for multifocal recurrence. Although complete resection in unifocal disease may lead to longer survival, outcomes are significantly worse in cases of multifocal relapse. Recent reports show a median survival of about 34 months for multifocal abdominal recurrence, decreasing to nearly 18 months when more than seven lesions are present, with no clear survival benefit even after complete resection (6,17,20). These findings challenge the idea that just being technically resectable justifies surgery. Therefore, multifocality is increasingly seen as an indicator of aggressive biology, and surgery in these cases is often more palliative than genuinely prognosis-altering, except in highly selected situations with specific goals (20,21).

A second controversy involves re-irradiation and, more broadly, the role of radiotherapy in recurrent disease. Preoperative, intraoperative, and postoperative approaches have all been studied, but the previously operated retroperitoneum presents a narrow therapeutic window due to bowel displacement and cumulative tissue tolerance. Techniques such as intraoperative radiotherapy or brachytherapy may enhance local control in selected patients, but they entail significant toxicity and are not standard practice (17,28). Similarly, there is no strong evidence to support routine adjuvant radiotherapy following resection of recurrent disease. Conversely, for unresectable or gross residual tumors, definitive radiotherapy with modern techniques might achieve local control beyond 12 months in selected cases, although optimal indications, target volumes, and doses remain uncertain (11,17,28).

In the palliative setting, surgery should be considered only when the objective is clear: symptom relief, prevention of imminent complications, or restoration of oral intake or bowel function, rather than prolonging survival. Although palliation can be achieved in many patients, it may come at a substantial cost. In the classic intra-abdominal sarcoma series by Yeh and colleagues, symptom improvement was reported in 71% of patients at 30 days and 54% at 100 days, with 29% morbidity and 12% postoperative mortality. These figures support careful selection, explicit goal-setting, and avoidance of liberal palliative laparotomy when the expected benefit is uncertain (44).

Timing of intervention remains one of the most practical controversies in recurrent RPS. Immediate surgery is reasonable for obstruction, bleeding, ureteral compromise, rapidly progressive symptoms, or anatomically threatening disease. In asymptomatic recurrence, however, a defined period of observation may better clarify growth kinetics, multifocality, and biological tempo. Reports associating surgery within 6 months of recurrence diagnosis with worse disease-free survival should not be read as evidence that early surgery is intrinsically harmful; they more likely reflect confounding by indication, since aggressive, symptomatic, fast-growing, or anatomically threatening tumors are preferentially selected for earlier operation. Future studies should therefore model surgery as a time-dependent treatment decision and adjust for tumor kinetics, symptom burden, and anatomical risk, rather than treating early surgery as a simple fixed exposure (20,21).

Finally, extended locoregional strategies such as cytoreductive surgery with HIPEC or intraperitoneal chemotherapy remain highly controversial. Evidence is limited to small, selected series and has not consistently demonstrated benefit over surgery alone. These approaches should not be considered standard of care and should be used only in exceptional cases, ideally within protocols at experienced centers with clear objectives and strict selection criteria (22).

Overall, controversies in recurrent RPS highlight the challenge of making high-stakes decisions without solid prospective evidence. Progress relies on shifting focus from just technical possibilities to therapeutic appropriateness, considering histology, disease-free interval, multifocality, tumor growth, realistic resectability, and patient-centered outcomes. Only by using this framework can futile interventions be minimized and meaningful benefits enhanced (6,17,20).

### Future perspective

Management of recurrent retroperitoneal sarcoma (RPS) is evolving from a focus on anatomy and resectability to an approach based on measurable biology, dynamic risk assessment, and adaptive decision-making. Recurrence alone does not determine prognosis, and histology-specific failure patterns make uniform treatment strategies ineffective and potentially harmful (1,2,17,18). The challenge is to identify who needs treatment, when to intervene, and how to monitor the disease without unnecessary diagnostic burden (19).

A major area of progress will be the integration of clinically meaningful biomarkers. In RPS, this means moving beyond diagnostic classification to molecular and immunophenotypic characterization with prognostic and therapeutic significance at recurrence. Subtypes such as well-differentiated and dedifferentiated liposarcoma already serve as models, as recurrent molecular alterations are increasingly linked to targeted therapies and eligibility for clinical trials (38,39). The goal is to incorporate these biomarkers into long-term decision-making, guiding surveillance intensity, surgical thresholds, and prioritization of systemic therapy in biologically high-risk relapse. Circulating tumor-based approaches could also become useful surveillance tools to predict progression, distinguish viable tumor from postoperative change, and monitor treatment response, although clinical adoption will require proof of benefit beyond imaging and resolution of technical limitations such as low tumor fraction and clonal heterogeneity (19,45).

Future research should move beyond broad precision-medicine rhetoric and focus on recurrence-specific, testable strategies. In WDLPS/DDLPS, this includes a rational evaluation of MDM2-and CDK4-directed therapies, both for unresectable recurrence and as preoperative disease-control strategies, where durable growth arrest may be more clinically relevant than radiographic response. Liquid biopsy platforms—including ctDNA, copy-number–based assays, and methylation or fragmentomic profiling—should be incorporated as exploratory endpoints in prospective cohorts. Their greatest value may lie in detecting molecular residual disease, identifying high-risk recurrence earlier, and monitoring pharmacodynamic response, while recognizing that some liposarcomas may be low-shedding tumors (38,39,45).

A second area of progress is the move from static prognostic models to dynamic, AI-supported predictive systems. Current nomograms and consortium-based tools establish baseline risk but do not adequately capture longitudinal tumor behavior (6,13). The next step is to combine clinicopathologic risk with dynamic factors—especially tumor growth kinetics—and imaging features through radiomics or deep learning. The goal is to generate clinically actionable estimates that clarify when salvage surgery may change the disease course and when biology makes intervention ineffective (14,20). For routine use, these systems will need external validation, interpretability in multidisciplinary practice, and evidence that they improve decision-making and outcomes beyond statistical performance (6,13,14,20).

Another area of progress is risk-adapted surveillance. Fixed follow-up schedules are becoming less effective. Surveillance uses resources, raises scan-related anxiety, increases cumulative radiation exposure, and may lead to premature intervention without clear benefit, especially in indolent disease (19). Future strategies should therefore be customized based on histology, grade, recurrence history, tumor kinetics, and patient preferences, with defined action thresholds (19).

A fourth priority is developing trials specifically focused on recurrence. Recurrent RPS remains underrepresented in prospective studies due to its rarity, heterogeneity, and the ethical challenges of randomizing surgery-based strategies. Progress will likely rely on international pragmatic trials that use histology-based stratification and clinically relevant endpoints, such as survival, durable local control, time outside the hospital, patient-reported outcomes, and cost-effectiveness (17,20,43). Including biomarkers and predictive tools as stratification factors or prespecified secondary endpoints will be essential for translating personalization into practice.

A feasible research roadmap should prioritize recurrence-specific evidence generation over a universal management algorithm. The first step is to establish an international registry based on common definitions of true recurrence, residual progression, multifocality, resectability, surgical intent, and functional reserve. This should be followed by prospective cohorts that incorporate baseline postoperative imaging, serial volumetric assessments, central pathology review, patient-reported outcomes, and biospecimen collection. Although broad randomization of “surgery versus no surgery” is unlikely to be feasible in recurrent RPS, pragmatic trials could address narrower, clinically relevant questions, including observation versus early intervention for asymptomatic, low-volume recurrence; local versus systemic treatment for oligoprogression; histology-specific systemic strategies; and ctDNA- or imaging-adapted surveillance. This staged approach is more realistic for a rare, anatomically heterogeneous disease and better aligned with how decisions are made in multidisciplinary practice.

Overall, future management of recurrent RPS will rely on a revised decision framework rather than a single innovation: using biomarkers to interpret biology, dynamic prediction to estimate risk over time, adaptive surveillance to minimize diagnostic harm, and recurrence-specific trials to establish evidence-based standards.

## Conclusion

Recurrent retroperitoneal sarcoma is not simply a uniform treatment failure, but rather reflects different tumor biologies. Management should therefore go beyond just anatomy and technical resectability.

Meaningful decisions require considering histology, disease-free interval, multifocality, tumor growth kinetics, functional reserve, and expected treatment burden. Salvage surgery is appropriate only when it provides a realistic oncologic benefit with an acceptable functional cost.

The goal is not to increase the number of interventions but to boost the share of interventions that are biologically justified, proportionate, and patient-centered.
